# Digital emergency routing: analysis of feasibility, utilization, and equity implications

**DOI:** 10.1186/s13584-026-00761-4

**Published:** 2026-05-07

**Authors:** Osnat Bashkin, Tamar Shalom, Ilan Yehoshua, Limor Adler

**Affiliations:** 1 Department of Public Health, Ashkelon Academic College, Ben Tzvi 12, Ashkelon, Israel; 2 Department of Health Systems Management, The College of Law and Business, Ramat Gan, Israel; 3 Health Division, Maccabi Health Services, Tel Aviv, Israel; 4 Department of Family Medicine, Faculty of Medicine and Health Sciences, Tel Aviv University, Tel Aviv, Israel

**Keywords:** Digital health, mHealth, Telemedicine, Emergency medicine, Digital literacy, Health equity, Health services research, Health policy

## Abstract

**Background:**

Digital emergency care applications offer potential to reduce delays, enhance triage, and improve care coordination, yet evidence remains limited on their real-world implementation at scale. Maccabi Healthcare Services developed Maccabi-RED, a mobile application allowing patients to request urgent community-based care as an alternative to hospital emergency department visits. This study examines the implementation and utilization of Maccabi-RED during 2020–2023, aiming to describe demographic and clinical characteristics of patients initiating emergency care requests, identify factors associated with request approval and successful routing to community-based care and examine healthcare utilization patterns following app-initiated requests.

**Methods:**

This retrospective study analyzed de-identified electronic health record data from Maccabi Healthcare Services, including all patient-initiated emergency care requests through the Maccabi-RED application between January 2020 and December 2023. The study included 94,795 requests from 77,508 patients. We extracted demographic and clinical variables and examine patterns of subsequent healthcare utilization in the week following app-initiated emergency care requests, comparing approved versus non-approved requests.

**Results:**

During the study period, 51.6% of requests were approved, resulting in urgent community clinic appointments. Service utilization increased substantially from 11,058 requests in 2020 to 36,532 in 2023. Approved requests were more common among older patients and those with chronic conditions. Emergency type strongly influenced approval rates, with foreign body cases showing substantially higher approval odds than orthopedic cases. Geographic, ethnic, and socioeconomic disparities in approval rates were observed. In adjusted analyses, approved requests were associated with lower 7-day healthcare utilization, including fewer primary care physician visits and reduced odds of hospital emergency department and emergency medical center visits.

**Conclusions:**

The Maccabi-RED application demonstrates feasibility of scaling patient-initiated digital emergency routing, with potential to reduce downstream acute care utilization. However, observed approval disparities across age groups, geographic regions, and socioeconomic strata indicate that digital maturity alone does not guarantee equitable access. These findings underscore the importance of embedding equity considerations in system design, monitoring protocols, and capacity planning. Future development, including artificial intelligence-enabled decision support, should prioritize transparency and algorithmic fairness to improve performance without amplifying existing health inequities.

**Supplementary Information:**

The online version contains supplementary material available at 10.1186/s13584-026-00761-4.

## Background

Digital technologies are reshaping healthcare delivery worldwide. Over the past two decades, electronic health records, telemedicine, mobile health (mHealth) applications and data-driven decision support have moved from pilot projects to core components of many health systems [1, 2]. Reviews of digital transformation in healthcare show consistent potential benefits across settings: improved access to care, more efficient resource use, and opportunities for more personalized, continuous, and patient-centred services. At the same time, these technologies introduce new challenges related to interoperability, data governance, workflow redesign, and unequal digital literacy among patients and clinicians [3, 4]. Recognising both the promise and the risks of this transformation, the World Health Organization (WHO) adopted a Global Strategy on Digital Health 2020–2025, recently extended to 2027 [5]. The strategy articulates a vision in which digital health solutions support universal health coverage, strengthen health systems, and promote health equity, provided they are implemented within robust national strategies that align technological, financial, organisational and human resources.

Emergency care has emerged as a particularly important domain for digital health innovation. Prehospital and emergency department (ED) settings are characterised by time-critical decision-making, high clinical complexity, and rising demand. Systematic reviews indicate that digital and mobile technologies in prehospital emergency care can enhance clinical decision-making, improve operational efficiency, and strengthen communication between emergency medical services (EMS) providers and receiving facilities [6, 7]. Within this broader digital emergency care landscape, mHealth applications occupy a central role offering a wide range of functionalities such as emergency alerting, disaster management, and prehospital support [8]. Prehospital emergency care is increasingly mediated by digital platforms that connect patients, dispatch centres, field providers and hospitals, with mobile applications often serving as the patient’s main interface with the system. Despite this rapid innovation, the literature also emphasises substantial implementation challenges. A review of digital transformation in healthcare systems identifies recurring barriers, including technological barriers, regulatory complexities, and the need for significant investment in infrastructure [9].

Evidence from international literature on EMS-activation and emergency mHealth suggests that such tools could reduce delays, improve accuracy of triage information, and strengthen continuity of care between prehospital and subsequent community or hospital services [10–12]. In parallel, the Internet of Things (IoT) has been defined as a paradigm in which heterogeneous “things” (e.g., sensors, RFID-tagged objects, and connected devices) are networked to enable identification, sensing, and data exchange at scale [13]. Early policy and concept-oriented work further described IoT as extending connectivity beyond traditional computers toward everyday objects that are readable, locatable, and addressable via the Internet, with Radio Frequency Identification (RFID) often highlighted as a key enabler [14]. Complementing these conceptual foundations, applied overviews have emphasized that IoT systems rely on devices equipped with data-capture and communication capabilities to transmit information over ubiquitous networks [15]. Within healthcare specifically, narrative reviews have suggested that IoT applications can support clinicians and patients by improving the availability and flow of information across care settings [16]. At the same time, the growth of IoT has been noted to generate large, complex data streams, motivating the use of data-mining methods and raising challenges related to analysis at scale [17]. Notwithstanding these developments, the evidence base remains limited regarding how large, digitally mature health maintenance organisations (HMOs) deploy and operationalise emergency-oriented mHealth, potentially complemented by IoT capabilities at scale. Less is known about how such applications are integrated into routine workflows, which populations adopt and use them, how they are utilised during real-world acute events, and whether their implementation is associated with meaningful improvements in process measures or changes in subsequent healthcare utilisation. Addressing these gaps is critical for policymakers and health-system leaders who must determine whether, and under what conditions, to invest in app-enabled emergency care pathways.

Against this international backdrop, Israel provides a distinctive context in which to study the implementation and impact of digital emergency care applications. The Israeli healthcare system offers universal coverage under the National Health Insurance Law and is organized around four nationwide, community-based HMOs that function both as payers and providers. The Israeli HMO model differs from traditional fee-for-service systems in ways that are particularly relevant to emergency care innovation. HMOs bear the financial risk for their enrolled populations, creating strong incentives to prevent unnecessary ED utilization and hospitalisations. This has spurred investment in alternatives to acute care, including 24/7 telephone consulting services, urgent care clinics, and more recently, digital triage and routing systems. At the same time, Israel’s relatively small geographic size, high rates of digital literacy (with smartphone penetration exceeding 90%), and health system characteristics, create favorable conditions for piloting, scaling, and rigorously studying data-driven interventions such as remote care, decision support, and personalized medicine [18].

Israeli HMOs have increasingly invested in consumer-facing digital services that extend access to care beyond traditional clinics and call centres. Research on Israeli health consumers’ attitudes toward mHealth indicates growing acceptance of app-based interactions alongside continued valuing of face-to-face care [19]. This acceptance of digital health interfaces provides a foundation for more complex applications, including emergency care routing systems. Maccabi Healthcare Services, one of Israel’s largest HMOs, has advanced its digital service delivery by embedding app-enabled workflows into care pathways. As part of this effort, Maccabi developed “Maccabi-RED”, an urgent-care routing service that operates through the Maccabi app and is intended as an alternative to unnecessary ED visits. The service can be activated by the patient, or by a primary-care staff (a physician or nurse), after which nearby community specialists receive a request and may accept the case for immediate evaluation and urgent care in the local community clinics (see Maccabi-RED app in Appendix 1). By facilitating rapid matching between urgent patients and available community clinicians, Maccabi-RED aims to shift appropriate cases from hospital EDs to community-based care [20]. To evaluate whether such routing services translate into meaningful short-term safety and quality outcomes, seven-day ED outcomes (e.g., ED revisits) are commonly used as a short-term quality and safety indicator because they capture early clinical deterioration, unresolved problems, and potential gaps in discharge planning and care transitions soon after an index ED visit [21]. Because this near-term window is actionable, it is frequently used in prediction and risk stratification work to support targeted follow-up and resource planning [22, 23].

Despite the international evidence suggesting potential benefits of emergency mHealth applications, and despite the rapid proliferation of such tools within Israeli HMOs, there are critical knowledge gaps regarding real-world utilization patterns when such systems are deployed at scale and insufficient understanding of which patient populations adopt and use app-based emergency care pathways, and whether adoption patterns differ in ways that could exacerbate or mitigate health inequities.

These gaps are particularly consequential in the Israeli context, where policy makers and health system leaders must balance competing priorities: managing costs and resource constraints, reducing ED crowding, maintaining or improving care quality and access, and ensuring equity across diverse populations.

The present study seeks to address these gaps by examining the implementation and utilization of Maccabi-RED within a large Israeli HMO during a four-year period (2020–2023). Specifically, the study aims to: (1) describe the demographic and clinical characteristics of patients who initiated emergency care requests through the Maccabi-RED app; (2) identify factors associated with request approval and successful routing to community-based emergency care; and (3) examine patterns of subsequent healthcare utilization in the week following app-initiated emergency care requests, comparing approved versus non-approved requests. By addressing these aims, we provide evidence relevant to health policy decisions regarding the scaling and governance of app-enabled emergency care pathways in Israel’s uniquely structured healthcare system, with implications for other integrated care systems considering similar innovations.

## Methods

### Study population and data setting

Data for this retrospective study were obtained from the EHRs of Maccabi Healthcare Services. We analysed all requests for emergency care made by patients through the Maccabi-RED app between January 2020 and December 2023 (*N* = 95,795 app requests). Emergency visits referred by a family physician or nurse to the Maccabi-RED service but not initiated by patients through the Maccabi-RED app, were excluded from the study. The data represented a geographically and demographically diverse population of Israel.

### Ethical considerations

This study was approved by the institutional review board of Maccabi Healthcare Services (0100-23-MHS). As a retrospective medical record review of de-identified patient data, no informed consent was required.

### Variables

For each Maccabi-RED request, we extracted the following demographic characteristics (Table 1): age, sex, socioeconomic status, sector (general population, orthodox Jewish, or Arab), residency (urban vs. periphery), smoking status, and comorbidities. We also retrieved data on the type of emergency care requested (orthopedics, foreign body, surgery, obstetrics, and gynecology).

Primary outcome variables included: (1) approved requests, calculated as the percentage of approved requests in which patients received scheduled RED visits within the specified time window; and (2) healthcare resources utilization following the patients request for emergency care, measured by subsequent family/pediatric physician visits, emergency medical center visits (visits in acute care center in the community), hospital’s ED admissions, and hospitalizations, within 7 days from the visit request (for any cause).

### Data analysis

We compared the characteristics of Maccabi-RED patients requests that were approved to those of Maccabi-RED patients requests that were not approved using a chi-square test for categorical variables and a two-sided t-test for continuous variables. In addition, logistic regression models were used to assess patient approved requests by demographic variables and to evaluate healthcare resource utilization by approved requests for RED visits. Data were analyzed using IBM SPSS Statistics 29.0 software.

**Fig. 1:**
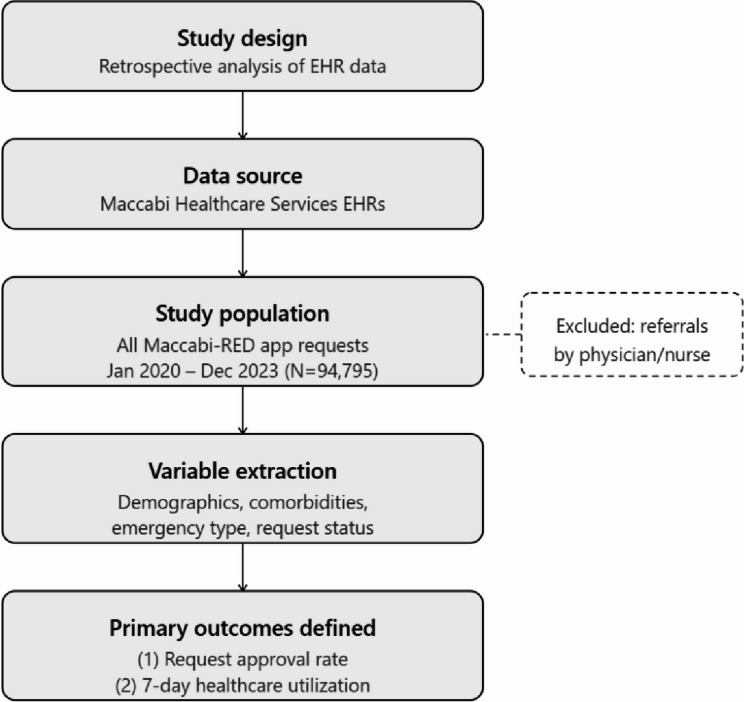
Maccabi-RED emergency care research stages

Figure 1 presents the research stages.

## Results

A total of 94,795 Maccabi-RED app requests for emergency care were recorded during the study period by 77,508 different patients. Of these, 48,972 Maccabi-RED requests (51.6%) were approved and received an urgent community clinic appointment at a community clinic.

**Fig. 2:**
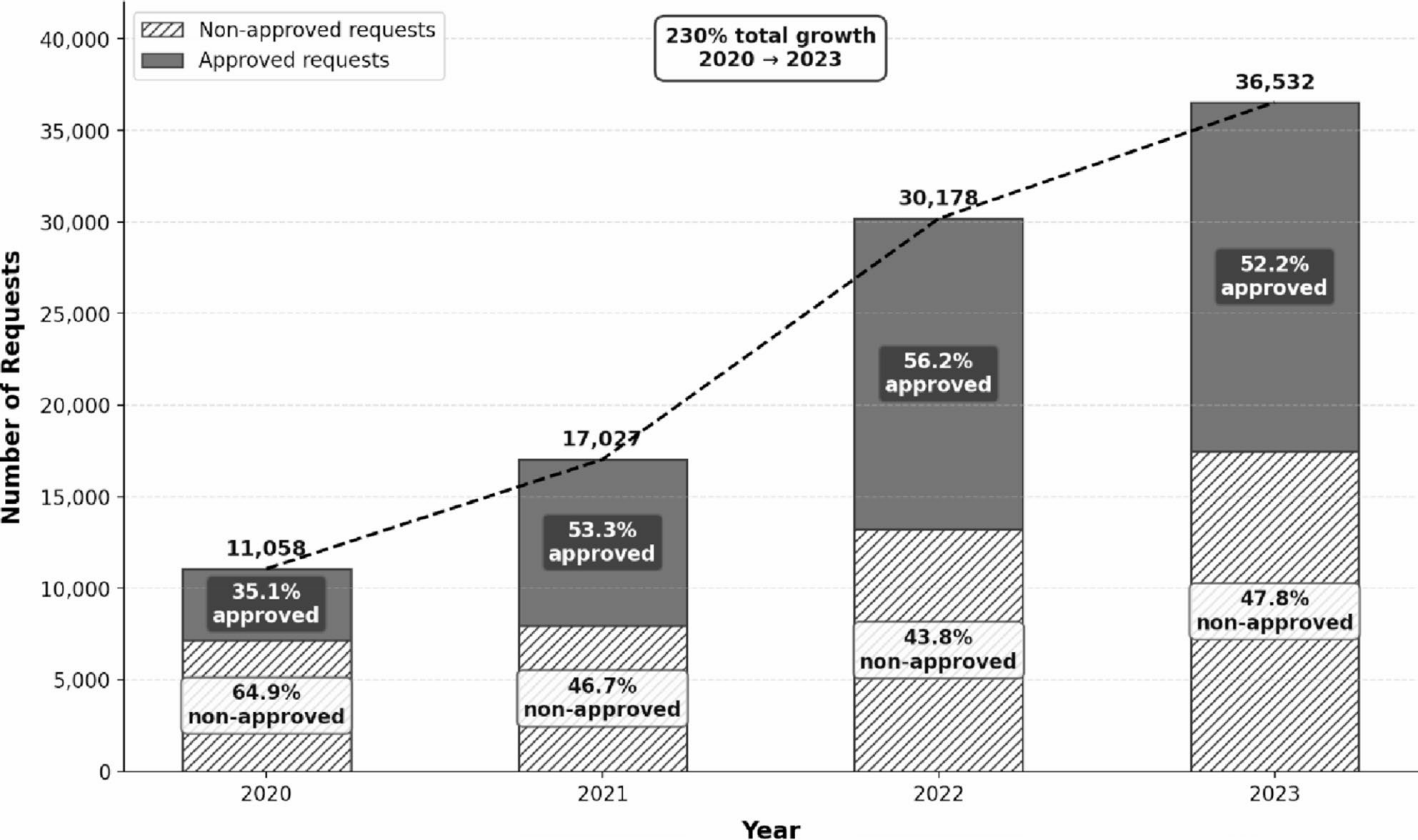
Temporal trends in Maccabi-RED emergency care approved and non-approved requests during 2020–2023

Figure 2 presents the temporal growth in Maccabi-RED service during four years of implementation. The service experienced substantial growth over the study period, with requests increasing from 11,058 requests in 2020 to 36,532 in 2023, suggesting rapid adoption during and following the COVID-19 pandemic.

**Fig. 3:**
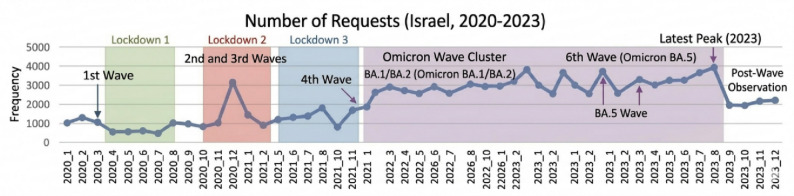
Number of requests across COVID-19 pandemic

Figure 3 presents the monthly frequency of Maccabi-RED requests in Israel during 2020–2023, mapped against the COVID-19 pandemic timeline including lockdown periods and waves. Request volumes were relatively low during the first wave and first lockdown (early 2020), followed by a notable peak during the second and third waves coinciding with Lockdowns 2 and 3 (late 2020 to early 2021). A sustained elevation in requests was observed throughout the Omicron Wave Cluster (BA.1/BA.2) beginning in early 2022, reflecting increased demand for community-based urgent care during this period. Request frequency remained consistently elevated through the 6th wave (Omicron BA.5) in mid-2022, reaching its highest point in mid-2023 before declining into a post-wave observation phase in late 2023. Overall, the figure illustrates that Maccabi-RED utilization tracked closely with pandemic activity, suggesting that COVID-19 waves were an important driver of app adoption and emergency care routing demand.

Table 1 presents the demographic and clinical characteristics of patients who requested Maccabi-RED requests, stratified by approved requests. Analysis reveals distinct demographic and clinical profiles between approved and non-approved Maccabi-RED requests, with several noteworthy patterns emerging from the 94,795 total requests. Approved requests were associated with older patients (mean age 37.3 vs. 32.8 years), with the > 51 age group comprising 30.0% of approved requests compared to only 20.4% of non-approved requests. Geographic disparities were evident, with peripheral area residents having lower approval rates (4.2% vs. 6.1% of requests). Ethnic patterns showed Arab patients had slightly higher approval rates (2.3% vs. 1.9%), while Orthodox Jewish patients had lower rates (3.5% vs. 4.0%). A striking clinical pattern emerged: patients with comorbidities had substantially higher rates of approved Maccabi-RED requests than Non-approved Maccabi-RED requests, including prediabetes (23.6% approved requests vs. 18.3% non-approved), hypertension (14.2% vs. 9.0%), cardiovascular disease (7.0% vs. 4.5%), and diabetes (7.1% vs. 4.5%). The type of emergency strongly influenced approval, with foreign body cases dominating approved requests (67.7% vs. 45.3%), while orthopedic (20.0% vs. 30.6%) and surgical emergencies (6.7% vs. 17.2%) had lower approval rates. These patterns suggest Maccabi-RED physicians may be preferentially serving older, chronically ill patients with foreign body emergencies, while younger patients and those with orthopedic or surgical needs face lower approval rates.

**Table 1:** Demographic and clinical characteristics of Maccabi-RED users by request status (*N* = 94,795)

	Maccabi-RED requests *N* = 94,795 (100%)	Non-approved Maccabi-RED requests *N* = 45,823 (48.3%)	Approved and scheduled Maccabi-RED requests *N* = 48,972 (51.7%)	*p*-value^1^
**Age in years at index date, mean ± SD and median [IQR]**	35.1 ± 21.1 33.0 [19.0–51.0]	32.8 ± 19.9 31.0 [17.0–47.0]	37.3 ± 22.0 36.0 [21.0–54.0]	< 0.001
**Age group**,** n (%)**				
< 19 years	23,507 (24.8)	12,080 (26.4)	11,427 (23.3)	< 0.001
19–33 years	24,046 (25.4)	12,928 (28.2)	11,118 (22.7)	
34–50 years	23,179 (24.5)	11,465 (25.0)	11,714 (23.9)	
> 51 years	24,062 (25.4)	9349 (20.4)	14,713 (30.0)	
**Area of residence**,** n (%)**				
Periphery	4875 (5.1)	2812 (6.1)	2063 (4.2)	< 0.001
Center	89,920 (94.9)	43,011 (93.9)	46,909 (95.8)	
**Ethnicity**,** n (%)**				
Arab	1987 (2.1)	846 (1.9)	1141 (2.3)	< 0.001
Orthodox Jewish	3514 (3.7)	1824 (4.0)	1690 (3.5)	
General	88,466 (94.2)	42,655 (94.1)	45,811 (94.2)	
**Sex**,** n (%)**				
Male	42,376 (44.7)	20,909 (45.6)	21,466 (43.8)	< 0.001
Female	52,418 (55.3)	24,912 (54.4)	27,506 (56.2)	
**Socioeconomic status**,** n (%)**				
Low	10,327 (11.0)	4902 (10.8)	5425 (11.2)	< 0.001
Middle	44,558 (47.5)	10,515 (45.3)	24,043 (49.5)	
High	38,928 (41.5)	19,835 (43.8)	19,093 (39.3)	
**Smoker**,** n (%)**	18,459 (19.5)	8757 (19.1)	9702 (19.8)	< 0.001
**Comorbidities**,** n (%)**				
Oncologic disease	4945 (5.2)	1949 (4.3)	2996 (6.1)	< 0.001
Cognitive impairment	934 (1.0)	312 (0.7)	622 (1.3)	< 0.001
Homecare	66 (0.1)	28 (0.1)	38 (0.1)	0.336
Prediabetes	19,951 (21.0)	8375 (18.3)	11,576 (23.6)	< 0.001
Hypertension	11,104 (11.7)	4144 (9.0)	6960 (14.2)	< 0.001
Cardiovascular disease	5466 (5.8)	2061 (4.5)	3405 (7.0)	< 0.001
Immunocompromised	1969 (2.1)	793 (1.7)	1176 (2.4)	< 0.001
Inflammatory bowel disease	1022 (1.1)	423 (0.9)	599 (1.2)	< 0.001
Chronic respiratory disease	1269 (1.3)	439 (1.0)	830 (1.7)	< 0.001
Chronic renal disease	2817 (3.0)	1022 (2.2)	1795 (3.7)	< 0.001
Diabetes	5549 (5.9)	2059 (4.5)	3490 (7.1)	< 0.001
Fall risk	6837 (7.2)	2221 (4.8)	4616 (9.4)	< 0.001
Hypercoagulability	179 (0.2)	62 (0.1)	117 (0.2)	< 0.001
Bone disease	5213 (5.5)	1873 (4.1)	3340 (6.8)	< 0.001
**Type of Emergency care**,** n (%)**				
Orthopedics	23,818 (25.1)	14,027 (30.6)	9791 (20.0)	< 0.001
Foreign body	53,928 (56.9)	20,769 (45.3)	33,159 (67.7)	
Surgery	11,149 (11.8)	7872 (17.2)	3277 (6.7)	
Obstetrics and Gynecology	5900 (6.2)	3155 (6.9)	2745 (5.6)	

*X*^2^ or T test

Table 2 presents the results of a multivariable logistic regression analysis for factors associated with requests approval (See detailed results of the analysis in supplementary file). After adjusting for all variables, several factors emerged as significant predictors. Analysis showed the service matured rapidly in its first two years, with 2021 and 2022 showing 93% higher odds of approval compared to 2020 (OR = 1.93, 95% CI: 1.83–2.03 and 1.84–2.02, respectively), though this advantage diminished somewhat by 2023 (OR = 1.54, 95% CI: 1.47–1.61), potentially reflecting capacity constraints as demand increased. Age-related patterns reveal that young adults (19–33 years) had the lowest approval odds (OR = 0.83, 95% CI: 0.80–0.87), while older adults (> 51 years) had significantly higher approval (OR = 1.23, 95% CI: 1.17–1.29) compared to children (< 19 years). Geographic and socioeconomic inequalities were found as peripheral residents faced lower odds of approval (OR = 0.83, 95% CI: 0.74–0.93), while high SES patients also had lower odds than low SES patients (OR = 0.82, 95% CI: 0.78–0.87). Interestingly, Arab patients showed higher approval odds (OR = 1.28, 95% CI: 1.16–1.42), while Orthodox Jewish patients had lower odds (OR = 0.89, 95% CI: 0.83–0.97). Among comorbidities, fall risk (OR = 1.28), inflammatory bowel disease (OR = 1.22), diabetes (OR = 1.17), and Hypercoagulability (OR = 1.18) emerged as significant predictors. Most strikingly, emergency type dominated all other predictors: foreign body cases had much higher odds of approval than orthopedic cases (OR = 2.17, 95% CI: 2.10–2.24), while surgical emergencies had lower odds (OR = 0.63, 95% CI: 0.60–0.66).

**Table 2:** Multivariable Logistic Regression Analysis of Factors Associated with Maccabi-RED Requests Approval

Variables	OR [95% CI]
**Index year**	
2020	Ref. (1.00)
2021	1.93 [1.83–2.03]
2022	1.93 [1.84–2.02]
2023	1.54 [1.47–1.61]
**Age group**,** years**	
< 19	Ref. (1.00)
19–33	0.83 [0.80–0.87]
34–50	0.97 [0.93–1.01]
> 51	1.23 [1.17–1.29]
**Peripheral residence**	0.75 [0.70–0.80]
**Sector**	
General	Ref. (1.00)
Arab	1.28 [1.16–1.42]
Orthodox Jewish	0.89 [0.83–0.97]
**District**	
Center	Ref. (1.00)
South	0.97 [0.92–1.01]
North	1.01 [0.96–1.07]
Sharon	1.03 [0.99–1.08]
Jerusalem	1.21 [1.16–1.26]
**Sex (male vs. female)**	1.02 [0.99–1.05]
**Socioeconomic status (SES)**	
Low	Ref. (1.00)
Middle	0.96 [0.91–1.01]
High	0.82 [0.78–0.87]
**Smoker**	1.00 [0.97–1.04]
**Comorbidities**	
Oncologic disease	1.00 [0.94–1.07]
Cognitive impairment	1.05 [0.90–1.21]
Homecare	0.79 [0.47–1.33]
Prediabetes	1.11 [1.07–1.15]
Hypertension	1.03 [0.98–1.09]
Cardiovascular disease	1.05 [0.98–1.12]
Immunocompromised	1.03 [0.93–1.14]
Inflammatory bowel disease	1.22 [1.07–1.39]
Chronic respiratory disease	1.09 [0.96–1.23]
Chronic renal disease	1.03 [0.94–1.13]
Diabetes	1.17 [1.09–1.25]
Fall risk	1.28 [1.19–1.38]
Hypercoagulability	1.18 [0.86–1.62]
Bone disease	1.04 [0.97–1.11]
**Emergency care specialty**	
Orthopedics	Ref. (1.00)
Foreign body	2.17 [2.10–2.24]
Surgery	0.63 [0.60–0.66]
Obstetrics and Gynecology	1.44 [1.35–1.54]

-2 Log Likelihood: 122088.71[S1.1]. Wald test for the overall model: χ² [34] = 7845.53, *p* < 0.001. Goodness of fit statistics: [S2.1] Cox & Snell R² = 8.0%. Nagelkerke R² = 10.7%. Overall classification = 62.3%

Table 3 examines healthcare utilization outcomes within 7 days of the Maccabi-RED request, comparing approved requests with non-approved requests.

**Table 3:** Healthcare utilization outcomes within 7 days following Maccabi-RED requests by appointment approval

Outcome noted within 7 days of the Maccabi-RED request		Non-approved Maccabi-RED requests *N* = 45,823 (48.3%)	Approved and scheduled Maccabi-RED requests *N* = 48,972 (51.7%)	Crude OR [95% CI]	Adjusted OR [95% CI] ^1^
Family physician/pediatrician visit	No	24,500 (53.5)	30,250 (61.8)	0.71 [0.69–0.73]	0.74 [0.72–0.76]
	Yes	21,324 (46.5)	18,722 (38.2)		
ED visit	No	45,675 (99.7)	48,869 (99.8)	0.65 [0.51–0.84]	0.67 [0.52–0.88]
	Yes	148 (0.3)	103 (0.2)		
Hospitalization	No	45,748 (99.8)	48,902 (99.9)	0.87 [0.63–1.21]	0.79 [0.56–1.11]
	Yes	75 (0.2)	70 (0.1)		
Emergency medical center visit	No	44,849 (97.9)	48,343 (98.7)	0.60 [0.54–0.66]	0.70 [0.63–0.78]
	Yes	974 (2.1)	629 (1.3)		

CI, confidence interval

^1^Adjusted for all variables included in the logistic regression in Table 2

Table 3 demonstrates that patients with approved Maccabi-RED requests had significantly lower subsequent healthcare utilization across multiple measures within 7 days, suggesting the service successfully diverts patients from traditional acute care pathways. Patients with approved requests had lower adjusted odds of visiting their family physician or pediatrician, with 38.2% requiring such visits compared to 46.5% of non-approved patients. Approved requests were associated with lower odds of ED visits (OR = 0.67, 95% CI: 0.52–0.88), though baseline ED utilization was very low in both groups (0.2% vs. 0.3%), and lower odds of emergency medical center visits (OR = 0.70, 95% CI: 0.63–0.78), where the absolute difference was more substantial (1.3% vs. 2.1%). These patterns suggest that Maccabi-RED approval and immediate community-based care may reduce the need for subsequent emergency care seeking, though the low absolute event rates and the inability to control for clinical severity limit causal interpretation. The fact that 38.2% of approved patients still saw their family physician within a week suggests either incomplete resolution of the presenting problem, new concerns emerging, or routine follow-up care.

## Discussion

This study examines the implementation and utilization of the Maccabi-RED innovative digital health app by analysing 94,795 app-based emergency care requests through Maccabi-RED over four years (2020–2023), of which 51.6% were approved for community-based care. The findings of this study have important implications for health policy decision-making in Israel and similar integrated healthcare systems. The substantial growth in Maccabi-RED utilization from 2020 to 2023 (231% increase) demonstrates feasibility of scaling app-based emergency care routing at the population level. Moreover, it may reflect both the pandemic’s push toward remote and digital care modalities and growing acceptance of such approaches in post-pandemic routine practice [24, 25]. The 2021–2022 period showed the highest approval rates (OR = 1.93 compared to 2020), potentially reflecting system optimization, increased clinician comfort with remote assessment, and expanded community capacity. The slight decline in approval odds by 2023 (OR = 1.54) may indicate capacity constraints as demand grew, or a shift in case mix as the acute pandemic subsided.

The association with reduced subsequent healthcare utilization, particularly for family physician visits and emergency medical center visits, suggests potential for cost savings and reduced pressure on acute care resources. However, the marked disparities in requests approval across demographic groups raise equity concerns that must be addressed before wider implementation. Three distinct barriers persist: patient-side barriers (difficulty using the app), system-side barriers (capacity constraints, algorithmic bias), and appropriate clinical prioritization. The higher approval rates among older patients and those with chronic conditions suggest clinical appropriateness drives much variation, while lower approval in peripheral areas points to capacity gaps. The findings highlight that even in digitally mature (widespread smartphone penetration of above 90%, robust electronic health record integration, and organizational readiness for digital service delivery), universal-coverage systems, equitable implementation is not guaranteed and requires deliberate design and monitoring.

While digital health interventions like Maccabi-RED offer transformative opportunities to advance health equity by overcoming geographic isolation, transportation difficulties, and limited appointment availability [26–29], they also risk exacerbating the digital divide if equity considerations are not embedded from the outset. The disparities we observed across age groups, geographic regions, and socioeconomic strata reflect broader structural inequities documented in digital health literature. Populations with limited access to technology, lower digital health literacy, or lower socioeconomic status may be systematically disadvantaged by app-based care pathways [30–32]. As healthcare systems become increasingly digitalized, it is imperative to prioritize health equity as a fundamental objective and reframe approaches to design, evaluate, implement, and scale digital health interventions [33–35].

The Maccabi-RED implementation shares features with emergency mHealth applications developed internationally. Recent studies highlight how targeted mobile functionalities can support emergency response and prehospital decision-making. A recent EMS-focused prototype integrated a one-tap in-app emergency call that simultaneously placed a standard emergency-number call while transmitting real-time geolocation and basic profile information to the call-centre interface, substantially accelerating location acquisition compared with conventional calls [11]. Complementarily, a prehospital triage mHealth application for paramedics enabled structured data capture, automated triage-level calculation using the Revised Trauma Score, and generation of structured case reports, with usability testing indicating high user acceptability [12]. These initiatives demonstrate the feasibility and user acceptance of emergency-focused mobile applications across diverse healthcare contexts, providing validation for patient-initiated and provider-facing emergency routing systems like the Maccabi-RED.

The implementation analysis of Maccabi-RED reflects the broader digital health innovation ecosystem in Israel. Over several decades, the Israeli HMOs have accumulated rich longitudinal electronic medical records, contributing to Israel’s reputation as a global “digital health powerhouse” and creating fertile ground for data-driven innovation that includes hundreds of startups, strong collaborations between HMOs, hospitals and technology companies and government programs designed to leverage routine health data for research and innovation [36]. This ecosystem has positioned Israel at the forefront of digital health research, with HMO databases contributing to both developing and studying digital health interventions: integrated data systems enable comprehensive evaluation of outcomes, large member populations support adequately powered analyses, and organizational commitment to innovation facilitates rapid iteration and improvement. Future research could also examine whether Internet of Things (IoT) capabilities via connected devices and real-time data exchange can complement app-enabled emergency care routing at scale [13]. Such infrastructures may strengthen information continuity across settings and support clinicians and patients through more timely, context-rich inputs [15, 16]. At the same time, the volume and complexity of IoT-generated data raise analytic and governance challenges that should be addressed when evaluating real-world impact and equity implications [17].

Future iterations of Maccabi-RED could benefit from integration of artificial intelligence (AI) and machine learning capabilities. A systematic review that included articles from 2018 to 2025 describes applications ranging from automated triage, prediction of clinical deterioration, and optimisation of dispatch and routing to decision support tools that integrate vital signs, free-text call descriptions and contextual data [37]. A scoping review of the literature on AI and machine learning in prehospital emergency care reported a wide range of use cases and reported that AI models outperformed clinical performance and non-AI-based approaches in most comparative studies [38]. Nevertheless, implementation would require careful attention to algorithmic fairness given the demographic disparities we observed, ensuring that AI-driven decision support does not amplify existing inequities. Embedding equity in digital emergency routing requires three core strategies: real-time monitoring dashboards tracking approval rates by demographic characteristics, proactive capacity planning that expands specialist availability in underserved regions before scaling digital access, and system design modifications such as visual aids for users with lower digital literacy. AI-enabled decision support must ensure, algorithmic fairness through pre-deployment bias audits, transparent model documentation, and continuous monitoring.

### Limitations

This study has several limitations. First, as a retrospective analysis of a single HMO’s data, the findings may not generalize to other healthcare systems with different organizational structures, payment models, or populations. Second, we could not assess patient satisfaction, clinical outcomes, or reasons for non-approval, which are critical for understanding the service’s overall value and equity impact. Finally, selection bias may exist as the study only includes patients who initiated requests through the app, excluding those initiated through nurse or physician, those without smartphone access, digital literacy, or awareness of the service.

### Policy implications and recommendations

Based on these findings, we offer recommendations for multiple stakeholders to advance equitable digital health implementation. First, HMOs should invest in peripheral region capacity expansion to match digital availability with clinical resources, implement multi-channel access strategies including phone-based and health worker-assisted pathways to prevent digital exclusion, create incentive structures for specialists serving peripheral regions, and develop targeted digital literacy interventions for underserved populations. In addition, healthcare system planners should embed equity monitoring from inception, conduct prospective equity impact assessments before scaling innovations, allocate resources for alternative access pathways, and establish learning networks across HMOs to share equity lessons.

## Conclusions

This study offers timely, Israel-specific evidence that may inform health policy on the scale-up of app-enabled emergency care routing within a universal, HMO-based system. The rapid growth in Maccabi-RED use and the association between approved requests and lower subsequent healthcare utilization suggest that community-based digital urgent-care pathways may help reduce avoidable ED demand and ease system pressures, although these findings should be interpreted cautiously given the large sample size and the possibility that some statistically significant differences may be small in practical terms. At the same time, approval differences by age, geography, and socioeconomic status may signal an equity risk that is directly relevant to national priorities, underscoring the need for policy frameworks that pair digital expansion with capacity planning, routine equity monitoring, and targeted strategies to help ensure fair access across diverse populations. Healthcare systems considering similar innovations should recognize that routine equity monitoring must be resourced and prioritized rather than assumed.

## Supplementary Information

Below is the link to the electronic supplementary material.

Supplementary Material 1.

Supplementary Material 2.

## Data Availability

All data were collected from the electronic health records of Maccabi Health Services. According to the Israel Ministry of Health regulations, individual-level data cannot be shared openly. Specific requests for remote access to de-identified community-level data should be referred to the Kahn Sagol Maccabi Research and Innovation Center, Maccabi Healthcare Services.

## Abbreviations

WHO: World Health Organization
ED: Emergency Department
EMS: Emergency Medical Services
HMOs: Health Maintenance Organisations
AI: Artificial Intelligence
