# Supplementary material for: Digital emergency routing: analysis of feasibility, utilization, and equity implications
Source: Isr J Health Policy Res. 2026 May 7;15:17. doi: 10.1186/s13584-026-00761-4 (PMC13151258; doi:10.1186/s13584-026-00761-4)

**Supplementary file 1:**

**Multivariable Logistic Regression Analysis of Factors Associated with Maccabi-RED Requests Approval.**


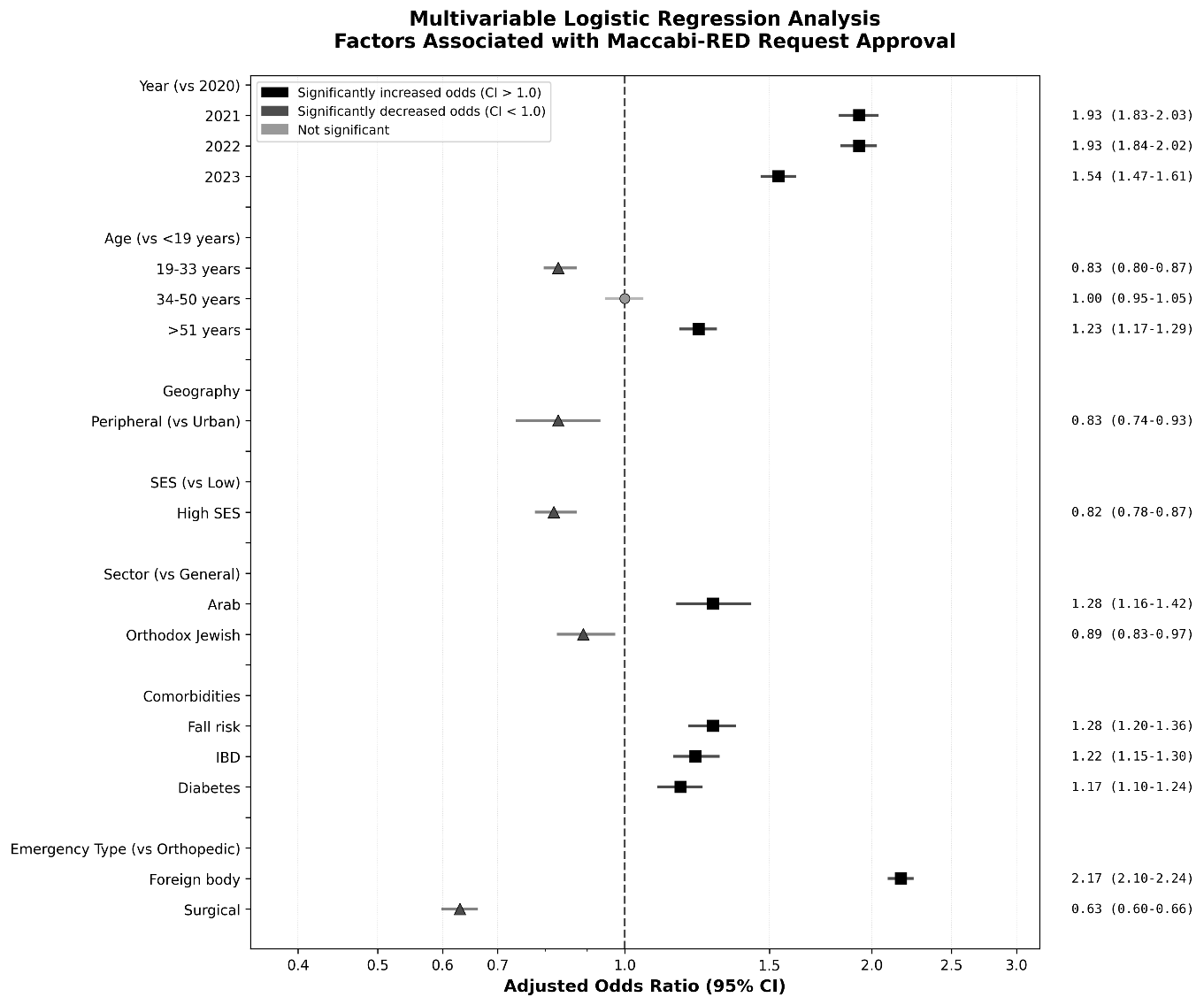

Supplement: Supplementary file 1 — Supplementary Material 1. [file 13584_2026_761_MOESM1_ESM.docx]
