# Supplementary material for: Digital emergency routing: analysis of feasibility, utilization, and equity implications
Source: Isr J Health Policy Res. 2026 May 7;15:17. doi: 10.1186/s13584-026-00761-4 (PMC13151258; doi:10.1186/s13584-026-00761-4)

**Appendix 1**

1. First screen of Maccabi application where the patient can find the option to request emergency care (pink button).


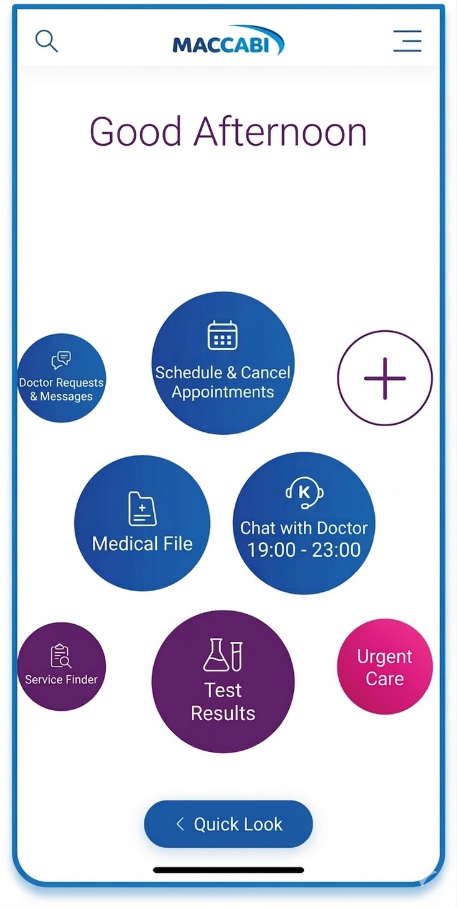


1. Second screen – entering the Maccabi-RED application.


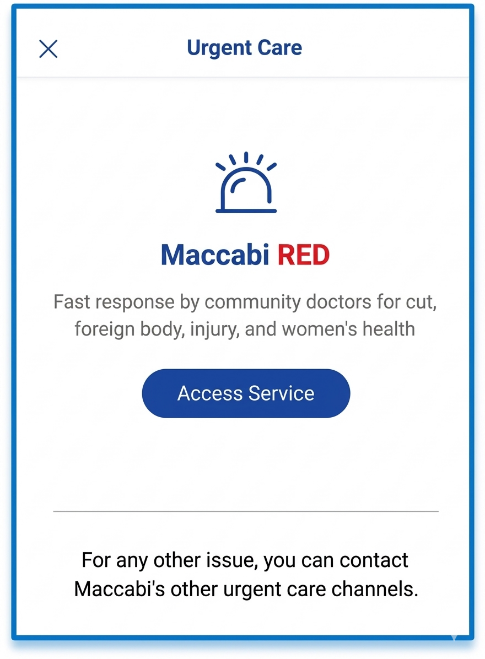


1. An example of a series of simple questions through which the Maccabi-RED application determines the correct referral for a patient - in this case following a foreign body injury.


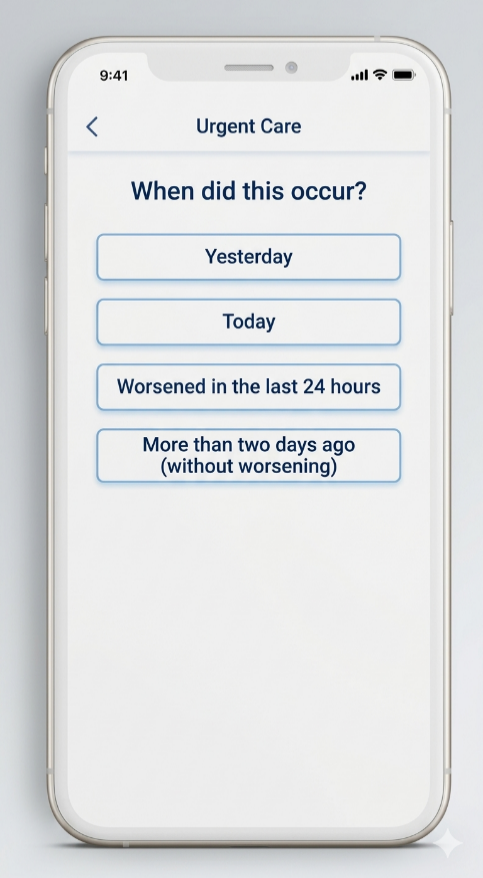


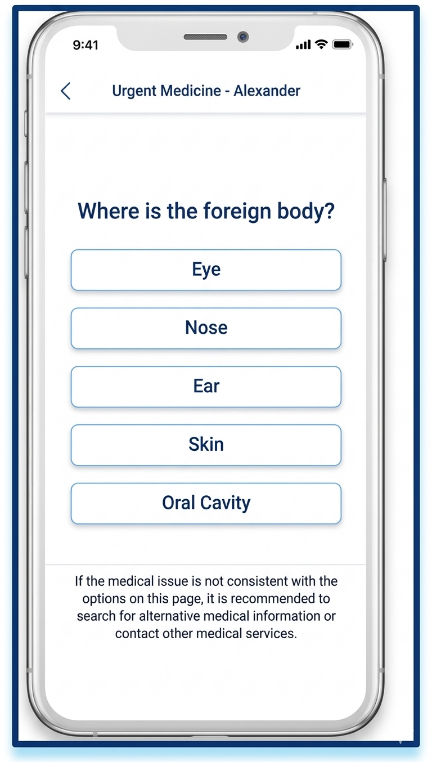


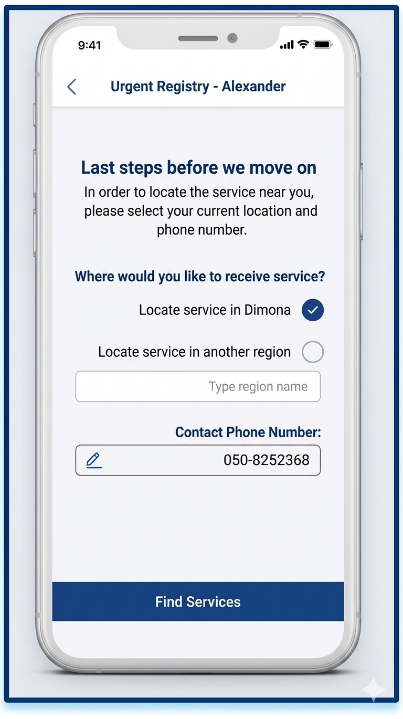


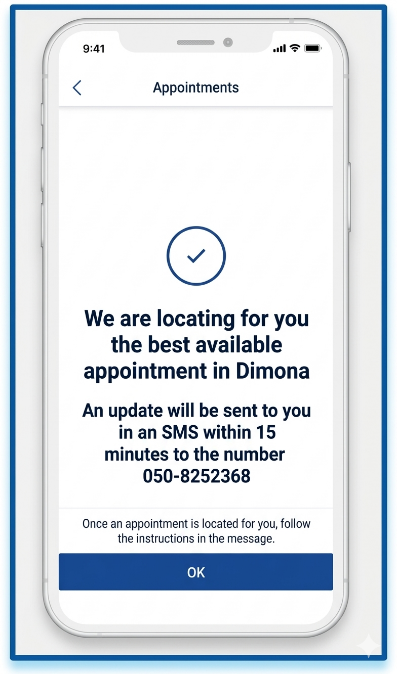


1. Possible decision screens in the Maccabi-RED application: 1. A message directing the patient to the first RED physician who answered the call, 2. A notification to the patient that he is near a Maccabi active care center that is available to treat him (RED service will not be activated), 3. The defined medical condition is not urgent and can wait for the attending physician.


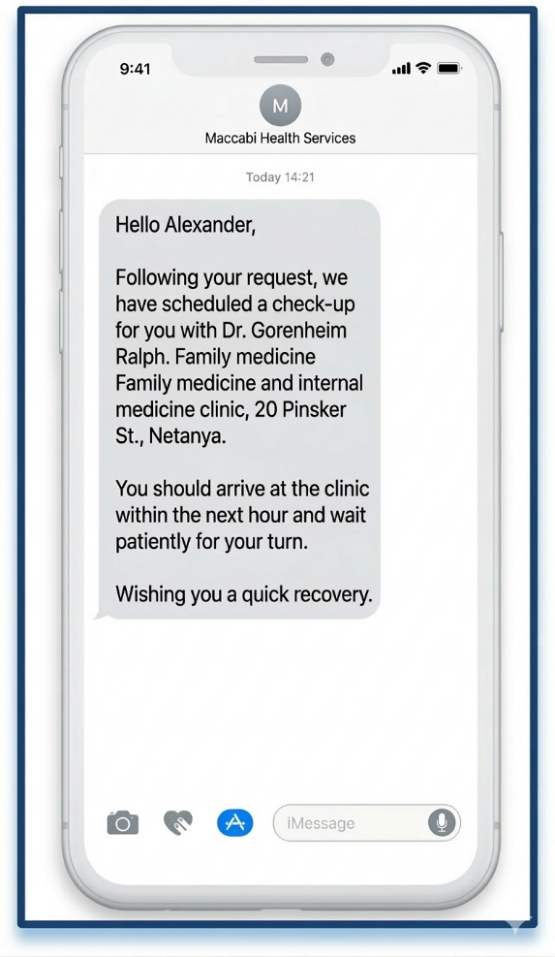


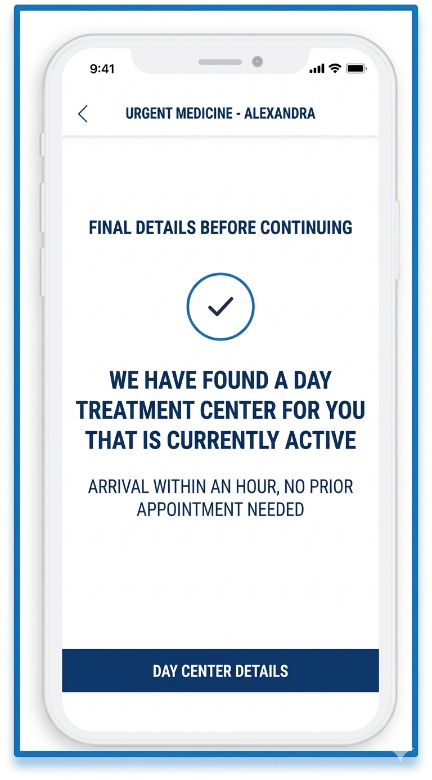


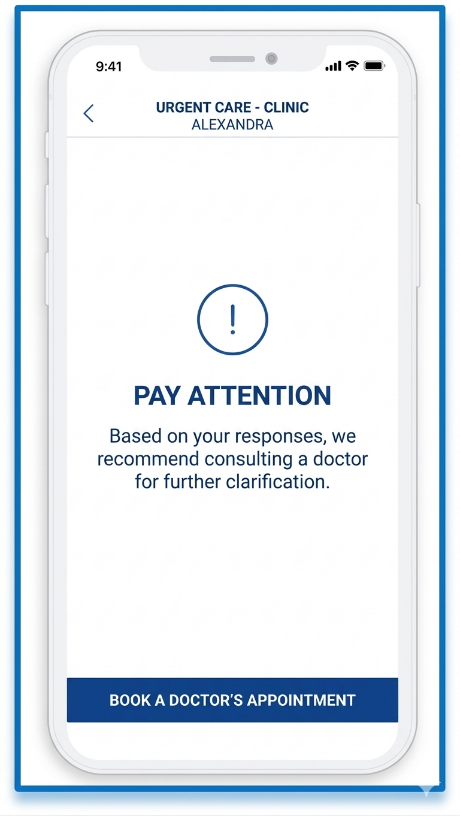

Supplement: Supplementary file 2 — Supplementary Material 2. [file 13584_2026_761_MOESM2_ESM.docx]
